# Advances in the Cystic Fibrosis Drug Development Pipeline

**DOI:** 10.3390/life13091835

**Published:** 2023-08-30

**Authors:** Christine Esposito, Martin Kamper, Jessica Trentacoste, Susan Galvin, Halie Pfister, Janice Wang

**Affiliations:** 1 Division of Pulmonary, Critical Care and Sleep Medicine, Donald and Barbara Zucker School of Medicine at Hofstra/Northwell, New Hyde Park, New York, NY 11042, USA; mkamper@northwell.edu (M.K.); jwang@northwell.edu (J.W.); 2 Division of Pediatric Pulmonology, The Steven and Alexandra Cohen Children’s Medical Center, Donald and Barbara Zucker School of Medicine at Hofstra/Northwell, Lake Success, New York, NY 11042, USA; sgalvin@northwell.edu; 3 Manhasset Office of Clinical Research, The Feinstein Institutes for Medical Research, Lake Success, New York, NY 11042, USA; hpfister1@northwell.edu

**Keywords:** cystic fibrosis (CF), cystic fibrosis transmembrane conductance regulator (CFTR), highly effective modulator therapy (HEMT), gene-based therapy, antibiotics, bacteriophage, anti-inflammatory, mucolytic therapy

## Abstract

Cystic fibrosis is a genetic disease that results in progressive multi-organ manifestations with predominance in the respiratory and gastrointestinal systems. The significant morbidity and mortality seen in the CF population has been the driving force urging the CF research community to further advance treatments to slow disease progression and, in turn, prolong life expectancy. Enormous strides in medical advancements have translated to improvement in quality of life, symptom burden, and survival; however, there is still no cure. This review discusses the most current mainstay treatments and anticipated therapeutics in the CF drug development pipeline within the mechanisms of mucociliary clearance, anti-inflammatory and anti-infective therapies, restoration of the cystic fibrosis transmembrane conductance regulator (CFTR) protein (also known as highly effective modulator therapy (HEMT)), and genetic therapies. Ribonucleic acid (RNA) therapy, gene transfer, and gene editing are being explored in the hopes of developing a treatment and potential cure for people with CF, particularly for those not responsive to HEMT.

## 1. Introduction

Cystic fibrosis represents one of the most striking examples of how the elucidation of the underlying pathogenetic mechanisms of an inherited disease has resulted in targeted therapeutics that have significantly altered the natural history of what, until recently, had been a relatively grim prognosis. CF is an autosomal recessive disorder which has been estimated to affect at least 100,000 individuals across the world [1]. The discovery of the culprit mutation of the cystic fibrosis transmembrane conductance regulator (CFTR) gene, which encodes the chloride ion channel responsible for transporting chloride and bicarbonate across the apical surface of secretory epithelia, has led to a remarkable improvement in the understanding of this multisystem illness. It is now estimated that, since the discovery of the original CFTR gene mutation in 1989, there have been over 2000 additional mutations characterized [1]. As is the hope with any disease process, with an improvement in the understanding of its pathophysiology there has been a resultant increase in the available therapies for people with CF (PwCF). While the foundation of treatment for CF will remain rooted in a team-based, multi-disciplinary approach that emphasizes thoughtful and selective use of anti-infective treatments, pulmonary hygiene, and adequate nutritional supplementation, among others, there is an ever-increasing array of therapies emerging which will not only enhance these well-established modalities, such as is the case with antibiotics, but will also introduce completely novel mechanisms of treatment. Although CF is and will continue to be a multisystem disease with significant morbidity, the abundance of treatment options either available now or in development represents a dramatic and optimistic shift in the outlook of a previously devastating diagnosis.

**Figure 1:**
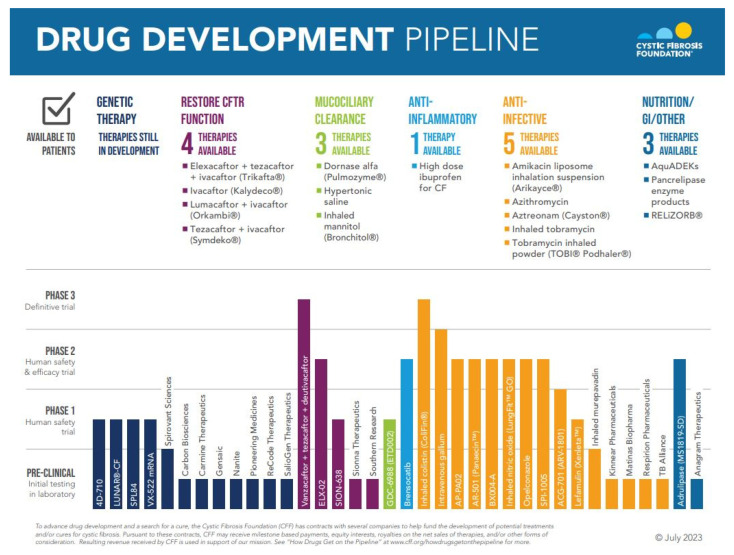
Reproduced with permission of the Cystic Fibrosis Foundation, Bethesda, Maryland. © July 2023.

The CF Drug Development Pipeline (Figure 1) has grown tremendously through the collaborative work between the Cystic Fibrosis Foundation (CFF), the medical and research community, and the pharmaceutical industry. The CFF’s commitment to accelerate drug discovery through venture philanthropy has led to significant gains in the care of PwCF, but it is far from complete. Here, we review impactful therapies in the areas of mucociliary clearance, anti-inflammatory, anti-infective, and restoration of CFTR function through modulator and highly anticipated genetic based strategies. Nutritional care is not discussed in this review, but we refer readers to an updated nutritional review by Frantzen et al. [2].

## 2. Restoration of CFTR Function with CFTR Modulator Therapy

CFTR modulators comprise a group of small molecules, grouped as potentiators or correctors designed to target the protein defects resulting from CFTR mutations [3]. Potentiators function by increasing the probability of the CFTR channel opening at the cell surface, thus increasing chloride transport and reducing the viscosity of exocrine secretions [4,5]. Ivacaftor (IVA) is an example of a potentiator that has effectiveness in patients with CF carrying a gating mutation responsible for abnormal closing of the channel. Among the multitude of mutations identified, G551D is a comparatively common mutation (affecting approximately 4%) which affects this ATP-dependent conformational change and, thus, limits CFTR mediated chloride transport. Although initial research confirmed the efficacy of IVA for this relatively common G551D mutation, subsequent work has established its efficacy in other gating mutations [4,5]. Unfortunately, gating mutations represent only one of five potential classes of CFTR mutations, and, thus, additional therapies have been developed to target this pathophysiologic diversity. The F508del mutation, which accounts for approximately 70% of CF mutations across the world, results in abnormal folding and trafficking of the CFTR gene to the cell surface and, thus, presents a problem not fully addressed with potentiators such as IVA [6]. IVA requires the presence of the CFTR protein at the cell surface in order to be effective. Thus, correctors were developed to target this deficiency in folding and to augment the amount of CFTR protein produced and available for further modulation. Correctors, when combined with a potentiator, result in clinically significant improvements in lung function in PwCF [7]. To date, commercially available combination therapies of corrector(s)/potentiator CFTR modulators include lumacaftor/ivacaftor (LUM/IVA), tezacaftor/ivacaftor (TEZ/IVA), and elexacaftor/tezacaftor/ivacaftor (ETI).

The efficacy of LUM/IVA was established in the TRAFFIC and TRANSPORT studies, two Phase 3 randomized controlled trials enrolling patients homozygous for the F508del mutation. Patients received the combination of LUM/IVA versus placebo for 24 weeks; the primary endpoint of the study was a change in forced expiratory volume in one second (FEV1) at the end of this period. In both studies, the patients who were randomized to receive LUM/IVA demonstrated improvements in their FEV1 versus placebo. Pulmonary exacerbations (Pexs) were also decreased in the treatment groups, as was the need for hospitalizations and intravenous (IV) antibiotics [7]. In a similar fashion, the EVOLVE study, a Phase 3 randomized controlled trial enrolling patients who were homozygous for the F508del mutation, demonstrated that TEZ/IVA improved FEV1 over a 24-week period in this population, as well as reduced rates of Pexs [8]. In addition to homozygous F508del mutations, a number of other mutations (A455E, D110E, E56K, etc.) have been determined to be responsive to these dual combination therapies, and possession of at least one of these mutations confers eligibility for treatment.

Following the dual combination therapies, triple therapy with ETI, or trade names Trikafta^®^ or Kaftrio^®^, was developed, consisting of two correctors combined with the potentiator IVA. The effectiveness of ETI was demonstrated in a Phase 3 randomized controlled trial in which change in FEV1 from baseline was evaluated in patients who were heterozygous for the F508del mutation and a minimal function mutation over a 24-week treatment period. Minimal function mutations, such as nonsense and splice mutations, do not encode for translation of a CFTR protein. Patients in this trial not only experienced improvements in FEV1 but were less likely to experience Pexs, require IV antibiotics or hospitalization, and reported improved Cystic Fibrosis Questionnaire-Revised (CFQ-R) scores, indicating higher quality of life. Although the overall rates of adverse events were similar between the treatment and control groups, patients assigned to receive ETI had higher rates of elevations in aminotransferase levels and development of rashes. The authors also reported higher rates of creatine kinase level elevations, as well as mild blood pressure elevations in the treatment, neither of which resulted in discontinuation of the drug [9]. Although generally considered well tolerated, other common side effects include abdominal pain and diarrhea. Baseline and periodic opthalmological exams are recommended in pediatric patients, given concern that IVA may increase the risk of cataract development in pediatric patients. ETI is currently approved for patients who have at least one copy of the F508del mutation and other mutations demonstrated to be responsive to this combination. These impactful medications are referred to as highly effective modulator therapy (HEMT) and have contributed to improvement in lung function as measured by FEV1, body mass index (BMI), respiratory symptoms (based on the CFQ-R respiratory domains), and a reduction in Pexs. As a result of HEMT, life expectancy is dramatically improving; the 2021 United States Cystic Fibrosis Patient Registry estimated that the median predicted age of PwCF born in that year was 52 years [10].

Research continues for the development of new HEMT with comparable or better efficacy and acceptable safety profiles (Table 1). One such example is a Phase 2 trial studying the safety and efficacy of a novel potentiator, deuticaftor (D-IVA or VX-51), a deuterated form of IVA that gives it greater stability, thereby increasing its half-life and achieving greater plasma concentration compared to IVA [11]. A Phase 3 trial studying vanzacaftor (VX-121), a novel corrector, in combination with TEZ and D-IVA in CF patients who are heterozygous for F508del and a minimal function mutation, is also currently underway [12]. Phase 2 studies using triple combination navocaftor (ABBV-3067), a potentiator, with the corrector galicaftor (ABBV-2222) and the addition of either experimental drug ABBV-119 or ABBV-576 in patients who were heterozygous or homozygous for the F508 del mutation had begun; however, these studies were discontinued for reasons yet to be released by the study sponsor at the time of this manuscript writing [13].

## 3. Genetic Therapies

### 3.1. RNA Based Therapies

**Figure 2:**
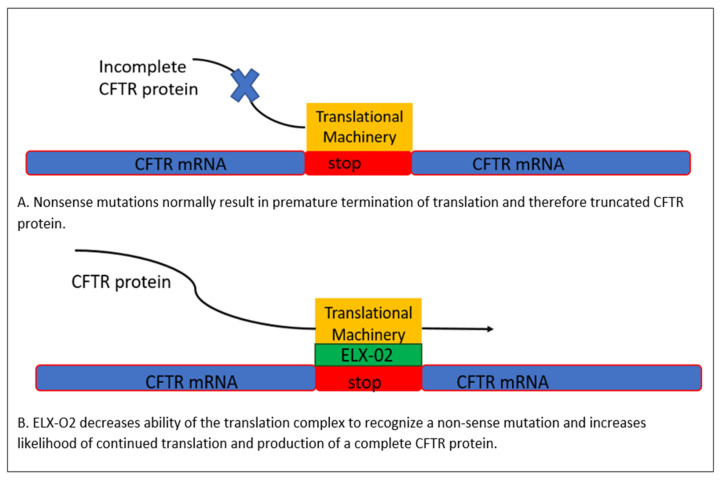
Under normal circumstances, translation is halted at stop or “nonsense codons” resulting in a truncated, non-functional CFTR protein. ELX-02 promotes continued translation in spite of such stop codons and increases the probability of full-length CFTR production [16].

In 2019, the CFF shared with the global CF community its research agenda, “Path to a Cure”, which aims to develop treatments and a cure for every person living with CF, while prioritizing those who are nonresponsive to current therapies. The focused strategies encompass CFTR protein repair and CFTR gene correction [14]. Although the effectiveness of CFTR modulators is encouraging with regard to the improved prognosis of most patients, the great pathophysiologic diversity of CF remains unaddressed in about 10% of PwCF who carry either minimal function mutations that are not known to be responsive to HEMT or unknown genotypes. For example, premature stop mutations lead to truncated CFTR proteins, which cannot be rescued by CFTR modulator therapies and represent a significant gap in currently available therapy [4]. Nonsense suppression, or readthrough therapy, seeks to suppress this premature termination [15]. Aminoglycoside antibiotics have previously been shown to correct this defect by promoting “readthrough”—that is, the CFTR protein is translated in spite of the premature stop mutation, enabling production of the full protein. Previously available aminoglycosides not only come with a host of known toxicities but have also not been demonstrated to have significant clinical efficacy. However, novel non-antibiotic glycosides, such as the compound dubbed “ELX-02”, have demonstrated the ability to promote readthrough of CFTR mRNA in the setting of nonsense mutations and their resultant premature stop codons, including the most common nonsense mutation, G542X, in human-derived “organoids” (three dimensional cellular structures derived from intestinal stem cells) (Figure 2) [16].

**Figure 3:**
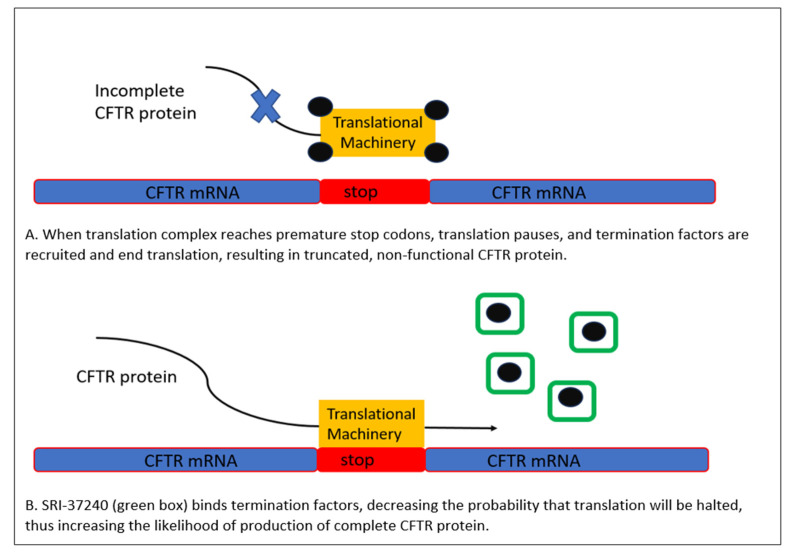
Mechanism of SRI-37240. By binding termination factors (black dots) and preventing their normal interaction with translational machinery at stop or “nonsense” codons, SRI-37240 (green boxes) increases the production of full length, and therefore fully functional, CFTR proteins [15].

Currently, a Phase 2 trial of subcutaneously administered ELX-02 in patients with CF with at least one G542X mutation is underway [17]. Current research is aimed at investigating the creation of novel aminoglycosides with more favorable side effect profiles and at novel strategies promoting CFTR translation. For example, recent research has identified another method with demonstrated ability to suppress CFTR nonsense mutations by promoting readthrough at premature termination codons via depletion of the termination factor, eRF1 [15]. Termination factors, as their name suggests, are proteins that mediate the termination of RNA transcription and may represent a novel target for future pharmacotherapy. Sharma et al., utilized CFTR nonsense mutation models derived from Fischer rat thyroid cells expressing human CFTR deoxyribonucleic acid (DNA) containing the G542X nonsense mutation and demonstrated that one identified compound, SRI-37240, not only possessed the ability to deplete eRF1 and increase mRNA production and CFTR function but also was selective for these nonsense mutations and did not interfere with suppression of normal termination codons (Figure 3) [15]. While compounds such as this are far from clinical application, they represent yet another promising method of restoring CFTR function and improving the lives of PwCF.

Aside from increasing CFTR mRNA with readthrough technology, another targeted therapy is introducing new, wild-type RNA into the cells to utilize in the production of complete CFTR protein. Research has encountered several challenges, which include ensuring adequate delivery of mRNA with effective penetration through the respiratory epithelium, as well as establishing an effective and practical dosing regimen to ensure sustained mRNA expression. RESTORE-CF, the first-in-human clinical trial of inhaled mRNA in PwCF, was a Phase 1/2 randomized, double-blind, placebo-controlled trial with the primary goal of evaluating the safety and tolerability of MRT5005, an mRNA delivered within a protective lipid nanoparticle aerosol [18]. The trial enrolled a total of 42 subjects with 2 class I and/or class II CFTR mutations over 3 parts: (A) single ascending doses (SAD), (B) weekly multiple ascending doses (MAD), and (C) daily dosing (DD) [18]. MRT5005 was, overall, well tolerated, with one serious adverse event being a pulmonary exacerbation. Similar to the observations of febrile reactions in mRNA vaccine trials against COVID-19, 14 mild or moderate febrile reactions were noted in 10 subjects, of whom 2 also developed hypersensitivity reactions, including urticarial rash. Although not a primary outcome, FEV1 improved in the SAD group, but this was not replicated in the MAD and DD groups, raising concerns over the lack of efficacy signals. The precise reasons for the FEV1 discrepancies have not been elucidated, although there are several possibilities. Firstly, FEV1 was frequently measured over a short timeframe and the observed increases in FEV1 may not have reflected the varying measurements that may occur later in time; once corrected, CFTR proteins were made and mucous clearance of the airways took place. A possible reason for this may be the poor uptake of the lipid nanoparticles, resulting in inadequate expression of CFTR proteins. Bronchoscopy was not included in the trial design due to risk–benefit assessment; thus, adequate MRT5005 uptake could not be confirmed. The self-limited bioactivity of mRNA itself may be another area to address, in relation to frequency and duration of dosing. The small cohort sizes and the inclusion of patients taking ETI could have, additionally, played a role in diminishing any effects on FEV1 [18].

Several other compounds in the pipeline also utilizing nebulization of CFTR mRNA encapsulated by a lipid nanoparticle include ARCT-032, ReCode, and VX-522 [19,20,21,22]. A Phase 1 trial investigating the safety and tolerability of VX-522 is underway in PwCF who have CFTR mutations not responsive to modulator therapy [23]. Preclinical data from the trials of ReCode and ARCT-032 therapies support advancement of the studies into the next research phase; ARCT-032 has recently been approved for first-in-human studies [20].

The epithelial sodium channel (eNAC) also plays a role in the pathophysiologic mechanisms of CF. In response to diminished and defective CFTR channels, eNACs are upregulated, which leads to increased absorption of sodium and fluid from airway surfaces and resultant thickened secretions, further impairing mucociliary clearance. Short-interfering RNAs (siRNAs) are RNA molecules that function as parts of RNA-induced silencing complexes that bind to and cleave mRNA, and they have been developed to target the mRNA responsible for the production of these eNAC channels [24]. In human CF bronchial epithelial cells and mouse lungs, siRNA delivery with nanocomplexes, which consist of cationic liposomes and peptides capable of self-assembly upon mixing with siRNA, led to reduced levels of eNAC mRNA [25]. Most importantly, the aforementioned study also demonstrated normalization of ciliary function, restoration of airway surface liquid (normally decreased in CF), and decreased mucus protein concentration in the treated cell models. In addition to confirming that the effect of siRNA is sustainable such that it could offer a realistic modality of therapy, future research will also likely focus on enhancing its delivery across airway epithelium. For instance, lipid-polymer hybrid nanoparticles with PEGylated shells were analyzed as another delivery method of siRNA-laden nanoparticles. Despite the successful creation of hybrid lipid nanoparticles, penetration through mucus layers was limited in environments in which certain mucinous proteins found in the sputum of CF patients were present. Furthermore, the addition of the lipid shell was also found to prevent penetration of the nanoparticle across cell membranes.

Technical challenges encountered in the basic science setting have persisted into the clinical arena as well, as demonstrated in the RESTORE-CF trial [18]. Although promising in theory, the difficulties of the aforementioned studies highlight the challenge in the creation of RNA delivery systems: not only must these vectors penetrate the thick mucus layers characteristic of the vast majority of airways of PwCF, but, once through, the vectors must be able to enter the airway epithelial cells to be effective. Furthermore, they must be well tolerated by patients and result in clinically meaningful improvements in patient outcomes [26]. Other likely issues that will be faced by this therapeutic modality include the establishment of feasible dosing regimens and the optimization of feasible delivery systems such as nebulization. It is encouraging to note that, despite these challenges, several therapies with likely varying formulations of CFTR mRNA sequence composition, stability, and lipid nanoparticle vectors are being developed and studied in the pipeline.

Finally, anti-sense oligonucleotides, or nucleotides designed to bind to specific mRNA sequences, are being evaluated in a Phase 1/2 trial under the name SPL-84. This nebulized therapy aims to correct the 3849 +10 kb C to T splicing mutation, which normally results in the degradation of CFTR mRNA and the production of truncated non-functional protein that is not rescued by currently available modulator therapy. Antisense oligonucleotide application has led to FDA approved treatments for spinal muscular atrophy and muscular dystrophy, and SPL-84 represents yet another example of the development of mRNA therapy to treat CF [27,28]. It is promising to know that more novel therapies extending beyond CFTR modulation are in the therapeutic pipeline (Table 1). These innovative treatments represent yet another encouraging step towards providing a treatment for all PwCF.

### 3.2. Gene Therapy

Gene therapy, like RNA-based therapies (Table 1), are at the forefront of the next generation of new CF treatments. Gene therapy encompasses (1) gene transfer, in which the corrected CFTR DNA is delivered to cells, leading to synthesis of functional CFTR protein; and (2) gene editing, in which the CFTR mutation is repaired and the corrected copy of the gene is permanent [28]. Gene transfer vectors have been the target of focused research in this field. Adenoviral vectors has been investigated, but they are limited by inefficient transfer of genetic material, thought to be due to the absence of the cocksackie-adenovirus receptor in human epithelial cells lining the airways, among other reasons [29]. As has been recently demonstrated, recombinant adeno-associated viral vectors (rAAV) have the ability to correct the CF phenotype in nasal mucosa from mice and human intestinal tissue, as evidenced by an increase in fluid and chloride secretion in these models [30]. rAAV vectors are protein shells containing the transferring DNA of interest, and they are essentially protein-based nanoparticles without the immunogenicity and risk of triggering an inflammatory response following a “traditional” adenoviral infection. Currently, an rAAV vector called 4D-710 is being evaluated in a Phase 1/2 trial in CF patients with bi-allelic CFTR mutations or a single mutation who are ineligible or intolerant of available HEMT. Future research will likely emphasize the use or development of novel capsids to enhance the delivery of the wild type CFTR gene. For example, a hybrid vector utilizing the viral capsid of the human Bocavirus-type 1, which has demonstrated tropism for human airway epithelial cells, and the genetic material of recombinant adeno-associated virus 2 can be used to infect the proximal airway cells of ferrets in order to deliver genetic material [31]. While this research is far removed from clinical application at this stage, it represents the successful creation of a model by which human Bocavirus-type 1 capsid-based vectors may be developed and studied with the goal of creating effective rAAV based therapies. Other vectors for the delivery of genetic material include lentiviral vectors and viral liposomal vectors. A Phase 2B trial in CF patients of various genotypes evaluating pGM169/GL67A, a nebulized liposomal vector carrying the CFTR gene, demonstrated an improvement in FEV1 compared to placebo by 3.7% predicted at 12-months follow-up [32]. While these results are not striking, they are supportive that there may indeed be a role for these gene therapies in CF. Challenges facing these potential therapies include developing effective vectors targeting airway epithelial cells, sustaining genetic expression in human hosts, and limiting detrimental immunogenic responses.

**Figure 4:**
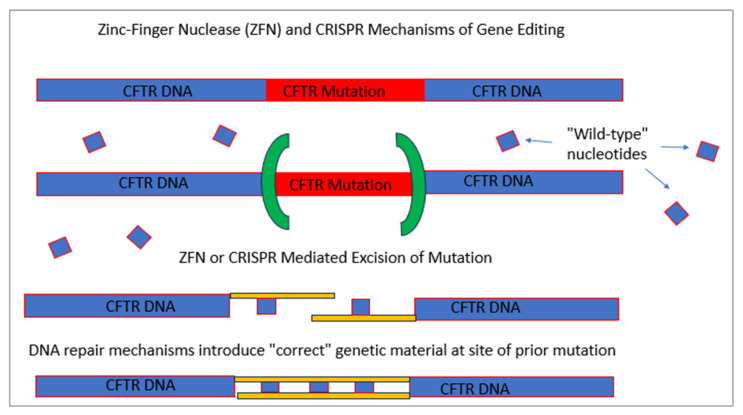
Simplified schematic of zinc finger nuclease (ZFN) and CRISPR/CAS9 technology: Both systems rely on the use of nucleases (green brackets) to remove mutations in the CFTR gene. Endogenous DNA repair pathways are then able to replace the removed genetic material with wild type nucleotides (small blue boxes).

Gene editing mechanisms aim to correct native host DNA. To this end, genetic therapies with zinc-finger nucleases (ZFNs), transcription activator-like effector nucleases (TALENS), and Clustered Regularly Interspersed Palindromic Repeats (CRISPR)/CRISPR-associated nuclease 9 (CAS9) operate under the principle that by creating double strand breaks into host chromosomes near the culprit mutation site, native repair mechanisms can then introduce and correct the functional genetic material initially absent (Figure 4). Crane et al. demonstrated that ZFN-mediated homology directed repair could be utilized to correct CFTR gene mutations in pluripotent stem cell lines derived from the skin fibroblasts of patients with CF who had one F508del mutation and one I507del mutation [33]. Restoration of proper CFTR expression and function through gene editing is an encouraging therapeutic approach toward curing CF; however, to target hundreds of pathogenic CF mutations is ambitious and not easily feasible. Furthermore, rarer mutations will take precedence due to the lack of any treatment targeting these genotypes. To address this issue, another study was able to successfully introduce a “super exon” into a cell line model with the F508del mutation, resulting in the transcription of “correct” CFTR mRNA [34]. Specifically, utilizing ZFNs, genetic sequences containing exons 11–27 of wild-type CFTR were inserted into the mutated cell line model, which was appropriately transcribed into functioning CFTR protein in the cells exposed to this therapy.

CRISPR/Cas 9 technology has also demonstrated the ability to introduce the correct F508del allele with restored CFTR function in adult intestinal stem cell lines with known F508del mutations [35]. Although these studies are promising given the potential of these various gene repair systems to restore native CFTR function in human models of CF, this therapy is based on introducing DNA strand breaks into native host DNA—an event that is, under normal circumstances, considered unwelcome and capable of significant collateral genetic damage. To circumvent this potential side effect, gene editing that does not depend on the action of nucleases has been investigated. For example, Mcneer et al. have shown that, in the absence of nucleases, peptic nucleic acids, which are synthetic oligonucleotide analogues, can be utilized to introduce donor DNA homologous to the CFTR F508del sequence in human bronchial epithelial cells and in the CF mouse model [36]. This confirmed that such a technique can restore wild type CFTR mRNA production and improve chloride ion efflux in treated human bronchial epithelial cells. Furthermore, histological and biochemical analysis did not reveal any increase in markers of inflammation within the mouse models, suggesting that this type of therapy may be of low immunogenic potential.

The current landscape of emerging therapy for CF is a dynamic and promising mix of strategies designed to target the disease at its most fundamental pathobiology. Established treatments of symptomatic consequences of defective CFTR function will remain a mainstay of care for patients with CF. However, the mechanisms described of restoring/augmenting CFTR function with small molecular modulators, amplifying functional CFTR expression with RNA based therapies, and genomic transfer and editing with restoration of “normal” sequences all provide hope that discussion of a cure should no longer be regarded as a far-fetched dream but rather a realistic goal within reach with advancing medical technology.

## 4. Antimicrobial Therapies

PwCF are frequently infected with a diverse range of opportunistic microorganisms that are naturally occurring in the environment and that can be challenging to treat. Impaired respiratory mucociliary clearance in CF leads to respiratory and sinus colonization, which contributes to Pexs, lung function decline, and chronic symptoms that impact overall health and quality of life [37,38]. The CFF recommends routine sputum surveillance to monitor for respiratory pathogens that are known to contribute to increased morbidity and mortality, with a focus on eradication and chronic treatment. Data from the United States CF Patient Registry in 2021 showed that *Staphylococcus aeurus* (SA) was the most prevalent pathogen amongst PwCF, occurring in 63.8% of patients, with methicillin-sensitive SA (MSSA) noted in more than half (51.7%) of individuals who provided a respiratory sample [10]. The prevalence rate of *Pseudomonas aeruginosa* (PA) has decreased notably over the last few years, from 46.4% to 28.4% between 2016 and 2021; this may be secondary to less sputum sampling from patients benefitting from HEMT and no longer expectorating sputum as much as prior to HEMT [10]. *Methicillin-resistant staphylococcus aeurus* (MRSA) was noted to be the third most common pathogen (18%) in 2021, mostly occurring in PwCF ages 10 to 20 years old. Many of these pathogens are known to cause a more rapid decline in lung health in PwCF [39]. Treatment of these pathogens should be individualized, with consideration focused on infection severity and chronicity, pulmonary function response, and tolerability of treatments with respect to medication-induced side effects and toxicities, allergies, and drug delivery methods [39]. The demand for novel antimicrobial treatments is growing with the increasing prevalence of multi-drug resistant (MDR) bacteria in the CF patient population. As PwCF frequently experience acute or chronic respiratory and sinus infections, their lifetime exposures to antibiotics leads to significant antimicrobial resistance and treatment challenges.

### 4.1. Oral and Intravenous Antibiotics

Antibiotic usage in the CF patient population is known to be high, given its mainstay role in treating acute Pexs, chronic respiratory infections, and optimizing respiratory health. Antibiotic administration may be oral or IV depending on the targeted pathogen, its drug susceptibilities, the severity of the patient’s lung disease, drug tolerance, and a multitude of other factors. Antibiotic choice is based on patient response and susceptibility testing, and recommended dosages are higher due to higher renal and non-renal clearance [40]. Newly established respiratory PA and MRSA infections are indications for eradication therapy [40]. Numerous studies have evaluated the efficacy of different regimens for PA eradication; the most commonly used drug is tobramycin inhalational therapy twice a day for 28 days [41]. The colonization of these organisms can lead to worsening lung function, progression of the patient’s symptoms, and lung disease. Aminoglycosides, fluoroquinolones, cephalosporins, trimethoprim-sulfa, tetracyclines, penicillin, and azithromycin are among the commonly utilized regimens for acute Pexs [42]. Anti-infective and anti-inflammatory properties unique to azithromycin were demonstrated in CF patients colonized with PA and with at least moderately severe lung function; thus, three-times-weekly use as an anti-inflammatory drug is standard of care for select patients [43]. Quite often, PwCF have more than one pathogen and will likely require antibiotics of different classes to improve coverage for Gram negative, Gram positive, and atypical bacteria. These higher doses and multidrug regimens can lead to increased side effects and reduced tolerability. NTM infections are another complex pathogen with challenges related to its lengthy treatment duration with a multi-drug regimen requiring close monitoring for intolerances and toxicities; this topic is beyond the scope of this review. The use of IV antibiotics to treat Pexs is very common. The STOP2 trial was the first study that sought to determine the optimal duration of treatment of Pexs in PwCF; it concluded that 10 days of IV antibiotic therapy is not inferior to 14 days, and, for those with less improvement after one week, 21 days is not superior to 14 days [44]. The CFF is also supporting the development of the STOP360 trial, which will evaluate if the addition of an IV aminoglycoside and IV beta-lactam antibiotic treatment is superior to the use of a beta-lactam alone to treat Pexs. This study hopes to show that monotherapy with an IV beta-lactam regimen is not inferior to a two-drug IV regimen, which would alleviate the use of aminoglycosides and its associated side effects of ototoxicity and nephrotoxicity [45].

### 4.2. Inhaled Antibiotics

The utilization of inhaled antibiotics has become a standard of care for the management of chronic infections in PwCF. Colonization and persistent airway infection can lead to inflammation of the airways, frequent Pexs, and pulmonary decline [46]. Inhalational delivery of medications into the lungs achieves higher drug concentrations directly into the target site of the small airways while minimizing the systemic side effects associated with oral and IV administration. Inhaled antibiotics have been shown to decrease bacterial load and, subsequently, airway inflammation and lung damage; they are also associated with improved clinical outcomes of FEV1 and reduced Pexs [47]. The most successful use of this treatment strategy has been in PwCF; however, it is important to note that there are a limited number of commercially available inhalational antibiotics [48]. The following qualities must be considered when choosing an antibiotic for inhalation: stability and tolerability, suitable pH, retained activity in the airway environment, and limited systemic absorption [49].

#### 4.2.1. Tobramycin inhalation solution

Tobramycin is an aminoglycoside antibiotic that is bactericidal against Gram-negative bacteria, including PA [39]. Tobramycin inhalation solution (TIS) is the best-studied aerosolized antibiotic for use in PwCF and chronic PA infections, and it is approved for people ages 6 and older [50]. TIS has been shown to be a safe and effective option for the treatment of PA, with improvement in FEV1 and a decrease in sputum pseudomonal density and hospitalizations [39]. Side effects of TIS include tinnitus, voice changes, and transient increases in creatinine, although long-term therapy has been proven to be well tolerated by CF patients with no unexpected adverse events [46]. In an effort to reduce treatment burden, as nebulization is time-consuming, a dry powder version of tobramycin (TOBI podhaler^®^) was approved for use in 2013. Controlled clinical trials have demonstrated that the tobramycin dry powder formulation has a safety and efficacy profile comparable to TIS with greater treatment satisfaction and adherence rates [46]. While eradication of PA is important, a majority of patients are either unable to clear the infection or have recurrent infection that becomes chronic, which is managed with on/off cycling of inhaled antibiotics every 28 days [49]. PA strains in CF tends to be mucoid, producing a thick biofilm that is protective against clearance and antimicrobial treatment strategies. In order to grow and multiply, PA has an absolute requirement for iron. The combination of tobramycin with calcium ethylene diamine tetra-acetic acid (CaEDTA) is currently being studied to improve the penetrability of the bacterial biofilms of PA [51]. CaEDTA is a chelating agent with antimicrobial properties; it binds to a variety of divalent cations including iron, zinc, and magnesium, rendering them unavailable for use by microorganisms. This phenomenon, referred to as “nutritional immunity,” has been successfully studied in vitro and in vivo in animals with chronic PA associated otitis, sinusitis, and endometriosis, and it weakens bacterial defense mechanisms, consequently increasing the bactericidal activity by the concomitant antibiotic [52]. Additionally, CaEDTA has been shown to disrupt the lipopolysaccharide layer in the outer membrane of PA, thus increasing the cell wall permeability and improving antibiotic penetration of the bacteria cell [52].

#### 4.2.2. Aztreonam Lysine (Cayston)

Aztreonam is a monobactam antibiotic that inhibits cell wall synthesis and is active against aerobic Gram-negative bacteria. Aztreonam lysine for inhalation (AZLI) is an aerosolized formulation that was specifically designed for inhalation therapy and is approved for people ages 7 and older who culture PA [39,50]. The safety and efficacy of AZLI for the treatment of PA has been demonstrated in a randomized, double-blind, placebo-controlled study. AZLI improved mean respiratory function scores and reduced sputum density of PA in patients with CF after 28 days of treatment with TIS as compared to placebo. In addition, AZLI increased the median time between Pexs that required antibacterial agents (AZLI, 92 days vs. 71 days in placebo; *p* = 0.007) [53]. In practice, depending on the severity of lung disease, TIS may be prescribed alone or alternating with another agent, such as AZLI, every other month. When following an “on” cycle use of a single inhaled antibiotic, a decline in lung function may be observed during the “off” cycle month. Alternating use of inhaled therapy with inhaled tobramycin and aztreonam, for example, can be more efficacious in patients with persistent respiratory decline [46,50].

#### 4.2.3. Liposomal Amikacin (Arikayce^®^)

Amikacin liposome inhalation suspension (ALIS) is currently approved for the treatment of refractory *Mycobacterium avium* complex (MAC). ALIS is therapeutically superior to amikacin alone, as the liposomes allow for the penetration and destruction of MAC biofilms, thereby achieving a higher therapeutic concentration [54]. Currently, ALIS is only approved by the U.S. Food and Drug Administration (FDA) for refractory MAC; however, it likely has utility in the treatment of other CF pathogens such as PA. Safety and tolerability of ALIS in PwCF who are colonized with PA has been studied and established globally [55]. In an open label randomized Phase 3 trial, cycling once daily ALIS proved to be noninferior to TIS for the treatment of chronic PA, as indicated by FEV1 improvements and reductions in PA sputum density. Patients also reported lower treatment burdens as measured by the Cystic Fibrosis Questionnaire-Revised (CFQ-R) [56].

#### 4.2.4. Colistimethate Sodium (Colistin)

Colistimethate sodium, a derivative of polymyxin, acts by disrupting the integrity of the bacterial cell membrane and is commonly used in PA infections in IV and inhalational forms [39,46]. Colistimethate sodium in dry powder formulation is approved in Europe and has been shown to be non-inferior to TIS for the treatment of chronic PA in patients with CF age 6 years and older [57].

#### 4.2.5. Inhaled Vancomycin

Vancomycin is a glycopeptide antibiotic that exerts its bactericidal effects by inhibiting cell wall synthesis, with activity against MRSA and other susceptible Gram-positive organisms [58]. Currently, there is no consensus guideline or recommendation for the treatment of MRSA infections in PwCF [39]. Inhaled Vancomycin was well tolerated and achieved good absorption in the sputum and high minimal inhibitory concentrations for MRSA in a Phase 1 trial [59]. Unfortunately, in a Phase 3 trial of the study drug (Aerovanc^®^), the primary endpoint of change in FEV1 was not reached and the trial was discontinued [60]. Preventing MRSA colonization through eradication attempts early in the newly acquired stage plays an essential role in delaying Pexs associated with MRSA [61]. Current ongoing trials are looking at combined PO antibiotics with nasal and throat decontamination as treatment approaches for MRSA eradication. The Staph Aureus Resistance-Treat Or Observe (STAR-too) protocol was the first randomized trial of eradication treatment for early MRSA infection in CF. The treatment arm received trimethoprim-sulfamethoxazole (TMP-SMX) and rifampin, combined with nasal mupirocin and throat decolonization with chlorhexidine, for 5 days and antiseptic skin washes for 5 days. Subjects also performed intensified environmental cleaning in their homes for 3 weeks. The control group was a no-treatment arm rather than a placebo arm, with a primary end point of negative MRSA culture on day 28 [62]. The study yielded positive results; however, there were concerns over the feasibility of the demanding treatment regimen and DDI with rifampin, which led to a simpler regimen proposed by the Staph Aureus Resistance-Treat Early And Repeat (STAR-ter) clinical trial [63]. STAR-ter is still currently enrolling subjects and using the same inclusion and exclusion criteria as STAR-too. In the STAR-ter clinical trial, subjects will be treated with one single oral antibiotic (TMP-SMX) and topical (nare and throat) decolonization. Subjects with an allergy or intolerance to TMP-SMX will use minocycline as an alternative antibiotic, and the primary end point remains MRSA-negative culture at day 28 [64].

#### 4.2.6. Inhaled Fluoroquinolones

Fluoroquinolones are bactericidal antibiotics that directly inhibit bacterial DNA synthesis [65]. They have been used extensively, as oral and IV formulations, to treat CF lung disease and Pexs. Fluoroquinolones have high potency and broad coverage, which makes them an attractive candidate for inhaled CF therapy [66]. Inhaled levofloxacin has been used for decades in the European Union and Canada for the treatment and management of PA [46]. It is well tolerated and associated with reduction in PA sputum load, FEV1 improvement, and decreased use of other anti-PA agents [66]. Although it did not meet its primary endpoint of reduction in Pex overall, it was still proven to be a safe and tolerable option for PwCF and PA infection of the airways [66].

### 4.3. Inhaled Murepavadin

Murepavadin is a pathogen-specific antimicrobial peptidomimetic that binds to the lipopolysaccharide transport protein in Gram-negative bacteria and causes cell death. It displays potent in vitro activity against PA in the IV formulation, and its application has recently been extended into inhaled therapies [67,68]. A Phase 1b/2a trial is planned to study inhaled murepavadin in adults with CF and is currently in the pre-clinical phase of investigation [69].

### 4.4. Phage Therapy

Research for new antimicrobials is a priority given the limitations in the currently available antibiotic treatments for PwCF, particularly in the setting of an aging patient population and high prevalence of MDR pathogens. Phage therapy utilizes viruses to infect specific bacteria and replicate the viral genome in the bacteria, ultimately lysing the host bacteria and killing it [37,70]. Phages, also known as bacteriophages, have been studied for decades, but they recently gained momentum in the CF research community because they may serve as an alternative treatment option with potentially fewer side effects for complex bacterial infections in PwCF [71]. Phages occur naturally in the environment, and they are found mostly in wastewater, reserves, lakes, and mammalian serum or compost [70,71]. They are currently being investigated for a variety of pathogenic infections in humans, including, but not limited, to sepsis, urinary tract, respiratory, skin, and joint infections [42]. The “ESKAPE” organisms (*Enterococcus faecium, Staphylococcus aureus, Klebsiella pneumoniae, Acinetobacter baumannii, Pseudomonas aeruginosa,* and *Enterobacter* species), along with other Gram-negative bacteria, have been designated priority pathogens by the World Health Organization against which to develop phages [42,71].

With limitations in the development of new antibiotics, phage therapy is currently being studied for patients with CF infected with PA, SA, *Achromobacter xylosoxidans*, and NTM, as seen in Table 2 [42,70]. In theory, phage therapy could be applicable for a multitude of pathogenic bacteria once an appropriate phage is identified, although mono-phage therapy may not be universally applicable to all patients with a particular pathogen. Bacterial resistance is ongoing, and phages will likely need to evolve as well [70]. Phage banks or libraries have been created and shared amongst scientists to expand access to and knowledge of existing phages [42,72]. Investigated phage delivery methods include oral, IV, and inhalational routes [42]. The AP-PA02 study utilized a phage cocktail against PA in vitro and demonstrated a reduction in over 80% of PA strains in patients with CF; this is currently under investigation in the SWARM-Pa study, a Phase 1b/2a trial for safety and tolerability [73]. In vitro and in vivo studies have demonstrated that phages also have the ability to adapt to bacterial mutations [70,72]. The BX004-A trial is a Phase 1b/2a double-blind placebo-controlled study investigating the safety and tolerability, as well as efficacy in reducing the PA bacterial load, of a nebulized bacteriophage for PwCF chronically colonized with PA (NCT05010577) [74]. Phages can penetrate the bacterial biofilms, which are not as easily penetrable by traditional antibiotics [71]. The use of single versus multiple bacteriophages in conjunction with antibiotics is also a strategy of interest, as this combination may have synergistic advantages in treatment [70].

Several published case reports have illustrated successful treatment with phage therapy in CF patients with infections from PA, SA, *Burkholderia dolosa,* and *M. abscessus* [37,75,76]. Reported outcomes following phage therapy included a reduction in sputum quantity and bacterial density, greater time between acute Pexs, and improvement in chest imaging, oxygenation, and weight gain [37,42,70]. A report of a pediatric CF patient with refractory respiratory symptoms and Pexs who was treated with inhaled pyophage, an inhalational preparation of five phages against PA, MSSA, *Streptococcus, Proteus*, and *E.coli*, demonstrated improvements clinically and in sputum bacterial density [75]. In another pediatric CF patient, nebulized pyophage against PA and MSSA was administered nine times at 4- to 6-week intervals with an interim addition of Sb-1 phage; this resulted in radiographic improvement and negative cultures [76].

Lung transplant CF recipients remain at risk for recurrent infections post-transplant, which poses an increased risk for chronic lung allograft dysfunction. Polypharmacy and drug toxicities in this patient population limits the use and duration of antibiotic regimens. Phage therapy has been used anecdotally in peri-transplant management of infections with promising outcomes [77,78]. In an adult CF patient with *B. dolosa* respiratory colonization following bilateral lung transplantation, recurrent pneumonias and postoperative complications led to the use of single lytic phage IV therapy for about 6 months in conjunction with IV antibiotics for 6 weeks [77]. Following phage therapy, clinical, radiographic and culture improvement was observed; however, due to liver failure, sepsis, and other complications, the patient died 11 months following transplant [77]. In a CF patient awaiting lung transplantation, a severe Pex led to endotracheal intubation, at which point a combination treatment with IV antibiotics and an IV cocktail of four lytic phages against MDR PA was given [78]. This patient was successfully extubated and underwent transplant at a later stage. In the case of a CF transplant recipient with a history of *M. abscessus*, the post-transplant course was complicated by disseminated mycobacterial infection, which was treated with a three-phage therapy via IV and topical routes for 32 weeks, with resulting respiratory and overall clinical improvement, weight gain, and surgical and skin wound healing [79]. All of these patients were reported to have tolerated phage therapy with no significant adverse events or side effects.

While there is encouraging research in phage therapy for CF patients, the clinical utilization of phage therapy is currently limited, and there is much to be learned about the safety profiles, potential side effects, tolerability, dosing regimens, costs, and need for re-treatment. Thus far, the reported adverse events have been few and not serious; however, there is still concern for potential allergic reaction during and following phage administration [37,42,70,71]. Current phage investigations are in preclinical or early clinical trial phases. The future of phage therapy could potentially help CF patients suffering from MDR infections. In the age of HEMT, many patients have experienced great benefits, such as reduced sputum production, making sputum sampling difficult and limiting monitoring of pathogens. It remains to be seen how far phage therapy will develop in the drug pipeline; however, it is a crucial area of development in the treatment of challenging infections and may even serve as personalized therapy specific to unique patients.

### 4.5. Other Antimicrobial Therapies

Other novel antimicrobial agents in the CF therapeutic pipeline include: IV and inhalational gallium, inhaled nitric oxide, sodium fusidate, lefamulin, and inhaled glycopolymer SNSP113 (Table 2).

Gallium is an element similar to iron that disrupts iron dependent processes necessary for bacterial survival and reproduction, and it has been demonstrated to be a safe and tolerable agent with associated improvement in lung function [80]. A Phase 2 study is currently evaluating the efficacy of IV gallium in CF patients chronically infected with PA (IGNITE study; NCT02354859) [81]. Also targeting PA infections is an inhaled formulation of gallium, AR-501 (NCT03669614) [80,82]. The ABATE study is underway, testing the safety and efficacy of 5 days of continuous IV Gallium in PwCF and *Mycobacterium avium* and *abscessus* infections (NCT04294043) [80,83]. A potential advantage of gallium therapy is its seemingly broad application against multiple pathogens.

Nitric oxide (NO) is being studied as an antimicrobial for its ability to break down biofilms and kill bacteria [84]. There are several clinical trials using NO in CF related NTM infections with refractory *M. abscessus* in the United Kingdom (NOMAB; NCT05101915), as well as MAC and *M. abscessus* in CF and non-CF patients in Australia (LungFit^TM^ GO; Beyond Air Inc., NCT04685720) [85,86,87]. Preliminary data from the LungFit Go study supported good tolerability of high dose NO in the hospital and home settings, as well as anti-inflammatory and pulmonary vasodilatory effects [84].

Sodium fusidate, also known as fusidic acid, is a protein synthesis inhibitor that prevents bacterial replication and has been studied as an antimicrobial against MRSA and PA infections in bone, joints, and skin; it is available in topical, oral, and IV forms, and it has already been approved for use in Europe [88]. Sodium fusidate is being further investigated in the U.S. for treatment of pulmonary SA infections, as monotherapy and in combination with other antibiotics [88]. The REPRIEVE study is currently investigating the efficacy and safety of oral sodium fusidate (ACG701) in CF patients with acute Pexs and receiving optimized antibiotic therapy (NCT05641298) [89].

Lefamulin (Xenleta^®^) is a pleuromutilin antibacterial compound that interferes with the bacteria’s ability to make protein during the translational process by binding to the bacterial ribosome and preventing the binding of transfer RNA for peptide transfer [90]. It is FDA-approved in oral and IV forms for the treatment of community acquired pneumonia caused by: *Streptococcus pneumoniae, MSSA, H. influenzae, Legionella pneumophila, Mycoplasma pneumoniae*, and *Chlamydophila pneumonia* [90]. Lefamulin also has antibacterial properties against MRSA strains obtained from PwCF [90]. A Phase 1 open label trial will be assessing the pharmacokinetics and safety of Lefamulin in adult CF patients with SA infections (NCT05225805) [91].

Inhaled glycopolymer SNSP113, also known as Poly-N-glucosamine (PAAG), disrupts biofilm and has anti-inflammatory properties in vitro; it is being investigated as monotherapy and with antibiotics to treat infections with SA, Gram-negative bacteria, and NTM [92]. Evaluations of the safety and tolerability of inhaled SNSP113 have been completed in Europe, and dose escalation studies are pending.

Opelconazole, an inhaled antifungal therapy, is being studied for the treatment of *Aspergillus* in PwCF [93]. Opelconazole is a synthetic triazole that acts to prevent the formation of the fungi cell membrane, resulting in lysis, and it is administered via nebulization at high concentrations with the goal of limiting systemic absorption, which can reduce many of the unwanted side effects of systemic antifungal medications [93]. There is an active open label study in the U.S. assessing the use of inhaled Opeconazole for 12 weeks as a prophylaxis or pre-emptive treatment of patients who have undergone lung transplantation [94].

**Table 2:** Antimicrobial therapies available and under investigation. ^1^ = therapies are commercially available; ^2^ = therapies are in clinical trial phase; ^3^ = therapies are in pre-clinical phase; ^4^ = commercially available in Europe. MRSA = *Methicillin resistant Staphylcoccus aureus;* MAC = *Mycobacterium avium* complex. Refs. [37,39,46,50,58,65,67,70,80,87,88,90,92,93].

Antimicrobial Therapies	Mechanism of Action	Targeted Pathogen
Inhalation Antibiotics		
Tobramycin ^1^	Aminoglycoside; inhibits protein synthesis of Gram-negative bacteria	*Pseudomonas aeruginosa*
Tobramycin dry powder ^1^		
Aztreonam ^1^	Monobactam; inhibits cell wall synthesis against aerobic Gram-negative bacteria	
Levofloxacin ^1^	Flouroquinolone; directly inhibits bacterial DNA synthesis	
Vancomycin ^1^	Glycopeptide; inhibits Gram-positive bacterial cell wall synthesis	MRSA
Colistimethate ^4^	Polymyxin derivative; disrupts the integrity of the bacterial cell membrane of most Gram-negative bacteria	*Gram negative*
Murepavadin ^3^	Antimicrobial peptidomimetic; binds to the lipopolysaccharide transport protein in Gram-negative bacteria and causes cell death	*Pseudomonas aeruginosa*
**Phage Therapy Studied Targets**		
*Pseudomonas aeruginosa* ^2^	Viruses that infect specific bacteria and replicate the viral genome, ultimately lysing the host bacteria and killing it	As noted
*Mycobacterium abscessus* ^3^		
*Staphylococcal aureus* ^3^		
*Achromobacter xylosoxidans* ^3^		
*Methicillin resistant Staphylcoccus aureus* ^3^		
**Other**		
Galium ^2^	Disrupts iron-dependent processes necessary for bacterial survival and reproduction	*Pseudomonas aeruginosa* MAC *Mycobacterium abscessus*
Nitric oxide ^2^	Gas compound that breaks down biofilms and kills bacteria	MAC *Mycobacterium abscessus*
Sodium fusidate ^2^	Protein synthesis inhibitor that prevents bacterial replication	MRSA *Pseudomonas aeruginosa Staphylococcal aureus*
Lefamulin ^2^	A pleuromutilin antibacterial compound that binds to bacterial ribosome, preventing the binding of transfer RNA and inhibiting the production of protein	MRSA *Staphylococcal aureus*
Glycopolymer SNSP113 ^2^	Disrupts bacterial biofilms; has anti-inflammatory properties	*Staphylococcal aureus,* MAC *Mycobacterium abscessus*
Opelconazole ^2^	Synthetic triazole; prevents the formation of the fungi cell membrane, resulting in lysis	*Aspergillus fumigatus*

## 5. Anti-Inflammatory Therapies

Chronic bacterial infections affecting PwCF trigger a chronic inflammatory state, and, while inflammation works to contain infection, it has harmful effects in the airways of PwCF [95]. In CF, the activation of toll-like receptor adaptor molecule (Trif)-dependent effectors are decreased, and this limited signaling interferes with the resolution of the inflammatory response, resulting in the inability to prevent the destruction of healthy lung tissue [96]. Studies conducted in infants with CF have shown evidence of inflammatory mediators present in the lungs before the detection of any signs of infection, suggesting that inflammation itself is intrinsic to the underlying pathophysiology in CF [97]. It is not entirely known why PwCF have chronic inflammation, but years of research has focused on some major contributors. Anion transport across CF airway epithelial cells is altered due to the defective CFTR protein [98]. As a result, there is a loss of chloride secretion leading to changes in osmotic pressure and electro-neutrality, which likely contribute to excessive sodium and water absorption [99]. The dehydrated airway surface liquid and mucus layer contributes to obstruction of the airway and chronic retention of pathogens, possibly serving as a trigger point for inflammation [98,99]. Additionally, there is an imbalance between omega-3 and omega-6 fatty acids, which may inhibit the capacity of cells to resolve inflammation [98]. Arachidonic acid (AA), derived from omega-6 fatty acids, is associated with the activation of an inflammatory response. In CF, AA levels are increased. Docosahexaenoic acid (DHA) is derived from omega-3 fatty acids and is associated with the termination of inflammation; in CF, DHA is reduced [98]. This relationship of higher AA levels relative to lower DHA levels in CF promotes the activation of inflammation. Furthermore, as part of the innate immune response, chemokines and chemoattractant molecules in the airways attract large quantities of neutrophils to clear the site of infection; however, neutrophilic phagocytosis is inefficient, particularly in excessive mucoid airways [100,101]. Neutrophils also release proteases, pro-inflammatory cytokines, reactive oxygen species, and neutrophil extracellular traps, thereby fueling further neutrophil influx and inflammation and ultimately causing lung damage [97,100,101]. Throughout time, clinical trials have been conducted to examine different therapies aimed at reducing inflammation in CF. Unfortunately, many anti-inflammatory trials have not produced a mainstay therapy to date.

The first anti-inflammatory drugs studied in CF were corticosteroids in the 1980s [95,99]. The first clinical trial of oral corticosteroids found that alternate-day prednisone dosing in children with CF ages 1–12 and mild to moderate lung disease was associated with better lung function, improved weight gain, and fewer hospital admissions [95,97,99]. Unsurprisingly, follow-up studies demonstrated increased adverse events with corticosteroid use, including glucose intolerance, growth impairment, and cataracts; thus, its use is not recommended for anti-inflammatory therapy in CF [97]. In 1994, a U.S. trial showed that twice-daily high dose ibuprofen of 200 mg was associated with slower lung function decline, better preservation of body weight, fewer hospital admissions, and no increase in adverse events in CF patients [95,97,98,99,102]. Ibuprofen is a non-steroid anti-inflammatory drug (NSAID) and inhibits neutrophil influx into sites of inflammation [97,99]. Although the results of this study led to the recommendation of ibuprofen for chronic use in CF, its use has not been widely adapted due to the challenges of obtaining a pharmacokinetic study to determine appropriate dosing and concerns regarding safety [97,99].

The use of a monoclonal antibody, KB001, was studied for its anti-inflammatory effects in CF patients with chronic PA infections. Although KB001 decreased sputum neutrophil activity, it was not associated with clinically significant outcomes in the frequency of Pexs or antibiotic need [98]. Inhaled Alpha-1-proteinase inhibitor (Alpha-1 HC), a highly purified blood product, was evaluated for its properties of reducing neutrophil elastase burden; however, it did not progress in trial phases [98,103]. A Phase 2 clinical trial of lenabasum, a cannabinoid type 2 receptor agonist that acts as an anti-inflammatory and anti-fibrotic agent, was conducted in PwCF and found to be associated with reductions in inflammatory biomarkers, IL-8, and immunoglobulin G; however, there was no significant change in FEV1, and a larger scale study remains to be seen [98,104]. Investigation of acebilustat, a leukotriene A4 hydrolase inhibitor that works by preventing the formation of LTB₄, a neutrophil chemoattractant, also resulted in the lack of significant differences in lung function or risk of Pex when compared to placebo [98,105]. While many of the aforementioned study drugs showed evidence of reducing inflammation, this did not translate into meaningful clinical outcomes as generally measured by frequency of Pex and lung function. In the era of HEMT, anti-inflammatory related outcomes may be even more difficult to capture, given the effectiveness of HEMT. Nevertheless, inflammation is still a major contributing factor to patients’ overall health.

New anti-inflammatory trials in the pipeline include several Phase 1 and 2 trials. A Phase 2 study using LAU-7b, a form of the retinoid-fenretinide, acts to increase DHA levels, thereby improving the AA/DHA imbalance found in CF and potentially reducing the inflammatory response in the lungs [98]. As mentioned, neutrophilic inflammation in CF is very characteristic of the inflammation in CF lungs and remains a crucial target for therapies. Brensocatib is an oral inhibitor of dipeptidyl peptidase 1, which is an activating enzyme of neutrophil serine proteases; brensocatib acts to inhibit the inflammatory cascade set off by neutrophils. Brensocatib was studied in non-CF bronchiectasis and found to delay time to Pex compared to placebo, and it may have applications in CF [106]. In Europe, a Phase 1 study is underway, evaluating the safety of inhaled lonodelestat (POL6014), which acts similarly to brensocatib by inhibiting neutrophil elastase [107]. Clinical development of anti-inflammatory drug therapies in the CF population is challenging due to a lack of validated and accepted biomarkers of drug efficacy and the need to study their effect on long-term clinical outcome measures [98]. Ongoing research that addresses these challenges is necessary in order to develop safe and effective anti-inflammatory drugs for CF.

## 6. Mucolytics and Mucociliary Clearance

Mucolytics and mucociliary clearance with accompanying use of airway clearance techniques has been essential in the care of CF patients, helping to clear the thick tenacious mucous that is a key factor of the disease. A major component of CF mucous is pus that is produced from viscous material derived from polymerized DNA from degraded neutrophils [108]. Finding effective mucolytics was among the first focuses of clinical trials supported by the CFF in attempts to break the cycle of pulmonary infection and inflammation from retained airway secretions. Dornase alfa (Pulmozyme^®^), the first drug targeted specifically for CF patients, was FDA approved for use in 1993 and remains a standard of care therapeutic in CF. Current CFF guidelines recommend the use of dornase alfa to improve lung function and reduce Pexs. Dornase alfa is inhaled via nebulizer once or twice daily and thins the mucous by acting like scissors to lyse through the sputum and by-products of inflammation in the airway. Sputum becomes easier to expectorate, airway clearance improves, and the risk of Pexs is reduced [109]. To date, three mucolytic agents have completed phase three clinical trials, been proven to be safe and effective, and are now available for patient use in the United States [110]. The second mucolytic approved for inhalation is hypertonic saline. Hypertonic saline works by increasing salt concentrations in the airway through osmotic forces, increasing the volume of the airway surface liquid. Clinical trials showed a short-term increase in airway surface volume associated with improved lung function and respiratory symptoms over baseline values [111]. This sterile solution, available in a 3% or 7% concentration, is inhaled via a nebulizer once or twice daily before airway clearance. The third and most recently FDA approved mucolytic therapy for PwCF 18 years and older is inhaled mannitol (Bronchitol^®^). Inhaled mannitol is a muco-active hyperosmotic agent administered as a dry powder with a hand-held inhaler that was shown to be an effective add-on to standard of care therapy in adults with CF to improve mucociliary clearance. The effects of inhaling mannitol were shown to be transient, so following the medication with airway clearance techniques, such as high frequency chest wall oscillation, is important to clear airway secretions [112].

In the current CFF drug development pipeline, trials for two additional mucolytic therapies include OligoG (AlgiPharma and CFF) and GDC-6998 (formerly EDT002) (Roche-Genetech and CFF) in Phase 1 trials. OligoG is in Phase 2 development, and it is a dry powder drug that has been shown to make mucous in the lungs thinner through calcium chelation, which is required for mucin unfolding and may help clear the airways of PwCF [113]. It has also been shown to increase the efficacy of some antibiotics. The second compound in the CF drug development pipeline for mucolytic therapy is GDC-6998 (formerly EDT002). GDC-6998 has been found to affect a chloride channel similar to the CFTR channel on the surface of airway cells by increasing the activity of this channel, TMEM16A. This may increase liquid surface fluid and improve mucous clearance [114].

The future of CF care guidelines in the U.S. is certain to undergo changes in the post-HEMT era. Along with the voices of patients in directing future clinical trials (CF Community Voice), the focus of clinical trials is also shifting toward reducing polypharmacy and care burden and improving quality of life [115]. The SIMPLIFY study evaluated the impact of discontinuing hypertonic saline or dornase alfa for six weeks, in PwCF with well-preserved lung function, and taking ETI. The results reported that discontinuing daily hypertonic saline or dornase alfa for six weeks did not result in clinically meaningful differences in lung function as measured by FEV1 [116].

## 7. Conclusions

Historically, CF therapeutics relied heavily on treating the disease progression after it had set in, as seen in antimicrobial and mucociliary clearance therapies. The field of CF treatments is moving further upstream in the disease process, as evidenced by HEMT, and it is now moving into mRNA and genetic-based therapies, which will help to address the remaining 10% of the CF patient population not eligible for HEMT. Drug development is also seeing more innovative approaches to tackle today’s challenges in CF disease. For instance, multi-drug resistant bacterial treatments are limited with today’s antibiotic choices, and more antimicrobial agents, such as bacteriophages, are needed outside of the traditional antibiotics. Another challenge is selecting measurable and detectable outcomes in the design of interventional drug trials. For instance, measures of FEV1 may not easily distinguish a detectable difference when the patient population is becoming healthier in lung function and overall health, leading the research community to consider the use of the lung clearance index through multiple breath washout testing as an alternative and more sensitive clinical trial endpoint [117]. As we move forward with the next wave of novel therapeutics, trial designs will need to evolve alongside the CF population. The future of CF therapeutics will undoubtedly have exciting advancements to benefit the growing CF population.

## Figures and Tables

**Table 1:** CFTR Targeted therapies. ^1^ = therapies are commercially available; ^2^ = therapies are in clinical trial phase; ^3^ = therapies are in pre-clinical phase; * Study was discontinued.

CFTR Modulators	mRNA-Based Therapies	Gene-Based Therapies
Ivacaftor ^1^	Aminoglycoside read-through nonsense mutation (ELX-02) ^2^	CFTR Gene Transfer Vectors: Recombinant adeno-associated viral vectors (rAAV) (4D-710) ^2^ Liposomal vector (pGM169/GL67A) ^2^ Lentiviral vector ^3^
Lumacaftor/Ivacaftor ^1^	Depletion of termination factor, eRF1 (SRI-37240) ^3^	
Tezacaftor/Ivacaftor ^1^	Inhaled CFTR mRNA (MRT5005 ^2^ VX-522 ^2^ ARCT-032 ^2^ ReCode ^3^)	
Elexacaftor/Tezacaftor/Ivacaftor ^1^	Short-interfering RNAs (siRNAs) ^3^	Zinc-finger nucleases (ZFN) ^3^
Deuticaftor (VX561) ^2^		Transcription activator-like effector nucleases (TALENS) ^3^
Vanzacaftor (VX-121)/tezacaftor/ Deuticaftor ^2^		Clustered Regularly Interspersed Palindromic Repeats (CRISPR)/CRISPR-associated nuclease 9 (CAS9) ^3^
Navocaftor (ABBV-3067), galicaftor (ABBV-2222) and ABBV-576 ^2^ *		

## Data Availability

Not applicable. No new data was created.
